# Report of Diseases Prevailing among Adults, from May to August 1822

**Published:** 1822-09

**Authors:** R. Macleod

**Affiliations:** Physician to the Westminster General Dispensary, &c. &c.


Art. II.—*Report of Diseases prevailing among Adults, from May to August* 1822. By R. Macleod, m.d. Physician to the Westminster General Dispensary, &c. &c.

In giving a report of prevailing diseases, it may not be foreign to the subject to take notice of the following passages, which occur in the Edinburgh Review for February 1822.

"While it is ascertained that marshy ground is the common cause of miasma or malaria, it is generally believed that either a considerable extent of such land, or a very decided marshy condition, is necessary to produce the disease. This, however, is a pernicious error, which it is important to correct.

"To recur to a very noted spot, however, it is not suspected that St. James's Park is a perpetual source of malaria, producing frequent intermittents, autumnal dysenteries, and various derangements of health, in all the inhabitants who are subject to its influence. The cause being unsuspected, the evil is endured, and no further inquiries made.

"It is lastly held, that it will not originate or spread in large towns; another dangerous opinion, leading to gross neglect in the treatment of diseases, and in the use of proper precautions against its effects. We have said that it is generated abundantly in St. James's Park, and thence it spreads even to Bridge-street and Whitehall: nay, in making use of the most delicate miasmometer (if we may coin such a word,) that we ever possessed, an officer who had suffered at Walcheren, we have found it reaching up St. James's-street even to Bruton-street, although the rise of ground is here considerable, and the whole space from the nearest water is crowded with houses. After this, we need scarcely remark that, at the east end of London, it reaches all through Finsbury division and Whitechapel, and is even brought up at the back of the Strand along the course of the river."

On perusing these extracts, any one, not acquainted with the circumstances, would naturally conclude that intermittents and autumnal dysenteries were the scourge of considerable portions of London; whereas, I have little hesitation in asserting that the evil exists only in the imagination of the writer. Indeed, the article seems to be the production of one entirely unacquainted with the diseases which prevail in London, however well he may know those of Rome. The discharge of my duties at the Westminster General Dispensary, has, for five years, called me almost every day to "the back of the Strand," and other parts of the town described as subject to the endemic prevalence of these effects of malaria, yet I have never met with any instance of these complaints; and it is obvious I must have done so had they existed. I have, besides, had an opportunity of observing the effects produced upon those very *miasmometers*, which the author in question regards as most perfect,—viz. persons who had suffered from the Walcheren fever. The regiments of Foot Guards are quartered, in rotation, in Lower Westminster, the part of the town most likely to be under the supposed influence of miasmata, both from the river and St. James's Park; yet it is not found that they suffer relapses from this cause. This assertion I make partly from personal observation, during eighteen months' attendance at the regimental hospital in Tothill-fields, but still more from the information of my friend and coadjutor, Mr. Bacot, whose experience is necessarily much more extended and convincing. In addition to these proofs, I have conversed on the subject with several practitioners of eminence; among others, with Dr. Baillie, who, as well as all the others, expressed the same opinion as I had myself entertained.

The character and respectability of the Journal which contains these doctrines have induced me to take notice of them, and, before concluding the subject, I beg leave to give one more extract: "But the east wind has the power of transporting it to considerable distances; and we have little doubt ourselves that, whenever it occurs in this city,

(where it is now rare,) the poison is transported from Holland."—It is impossible not to smile on perusing the formal and authoritative manner in which this opinion is delivered: in opposition to it, I would remark that, if the east wind does really bear the miasmata from Holland over so many miles of sea, it drops the burden before it arrives in London; for, during nine months' residence in the Tower, which, from its being situated at the east end of the town, and its proximity to the river, must have been exposed to the first visit of the malaria, not one instance occurred either of original attack of intermittent or of relapse; although I was in medical charge of a battalion of Foot Guards, at that time a regiment of "miasmometers."

During the last three months, fevers have been very mild in character and favourable in their termination. Cases of cholera have occasionally presented themselves, and have invariably done well under the use of diluents and opiates. Similar treatment, and with similar result, has been employed in the cases of diarrhœa.

In the case of diabetes, marked in the list of diseases, the urine was considerably charged with saccharine matter, and had increased in quantity to twenty pints a-day. The patient, a man about forty, was put upon animal diet, his meals consisting of beef-tea and meat without any vegetables; and, from his having been resident in St. Martin's workhouse, I have reason to believe the regimen was pretty strictly observed. In consequence of great restlessness, sleep was procured either by opium or hyoscyamus, according to the state of the bowels. Under this treatment, his strength was much improved, and the quantity of urine voided in twenty-four hours was reduced to seven pints by the end of about six weeks, when he discontinued his attendance.

A case of secondary small-pox occurred in a young man, who bore marks of having had the disease at a former period,—according to his own account, twenty-four years before. The eruption on the third day, when I first saw it, was pustulo-vesicular, and I was led, from this appearance, to expect that the disease would anticipate in its progress, in the same manner as small-pox after vaccination; but in this I was mistaken, for the pustules did not begin to dry before the eighth day, and the latter period of the eruption resembled that of common unmodified small-pox.

An opportunity has presented itself of trying the carbonate of iron in neuralgia: the result proved favourable. A young woman complained of violent pain in the face, which, upon further inquiry, was found to be aggravated at irregular times of the day and night, and to begin at particular spots. On being requested to point these out, she applied her fingers to the infra-orbitary and mental foramina of either side, indicating the exit of the nerves with a precision that would not have disgraced a pupil of Great Windmill-street. She took ℈j. of carbonate of iron three times a-day, under which treatment she gradually recovered, and was almost free from pain at the end of a fortnight, at which time she went into the country.

*Table of Medical Cases admitted, by* Dr. MACLEOD, *at the Westminster General Dispensary, from the* 1*st of May to* 10*th of August,* 1822.

**Diseases affecting particular Organs.*

| | | | |
|---|---|---|---|
| Head and Nervous System, in general | | Apoplexy | 1 |
| | | Determination to the Head | 14 |
| | | Paralysis | 2 |
| | | Epilepsy | 3 |
| | | Hysteria | 7 |
| | | Tremors | 2 |
| Particular Nerves (of Face) | | Tic Douloureux | 1 |
| Nostrils and Fauces | | Epistaxis | 2 |
| | | Catarrh | 11 |
| | | Cynanche Tonsillaris | 5 |
| | | Chronic Ulcers, with Eruptions | 5 |
| Organs of Respiration | | Laryngitis | 1 |
| | | Bronchitis | 13 |
| | | Pneumonia } | 9 |
| | | Pluritis } | |
| | | Hæmoptisis } | 18 |
| | | Phthisis } | |
| Organs of Circulation | | Pericarditis | 1 |
| | | Aneurism of Aorta | 1 |
| | | Palpitation, apparently from organic disease | 2 |
| Organs of Deglutition | | Stricture of the Œsophagus | 1 |
| Organs of Digestion | Stomach | Dyspepsia, Simple | 38 |
| | | ——— with Vomiting | 2 |
| | | ——— with Gastrodynia | 8 |
| | | ——— with Pyrosis | 2 |
| | | ——— with Hæmatemesis | 3 |
| | Bowels | Constipation, Simple | 10 |
| | | ——— with Colic | 8 |
| | | Colica Pictonum | 3 |
| | | Diarrhœa | 11 |
| | Liver | Hepatitis, Acute | 2 |
| | | ———, Chronic | 5 |
| | | Icterus | 2 |
| | Affecting all three, | Cholera Morbus | 7 |
| Organs of Urine | | Nephralgia | 1 |
| | | Diabetes | 1 |
| | | Dysuria | 11 |
| Organs of Generation | | Dysmenorrhœa | 1 |
| | | Amenorrhœa | 6 |
| | | Menorrhagia | 4 |
| | | Leucorrhœa | 7 |
| Skin—Eruptions | Acute | Erysipelas | 1 |
| | | Scarlatina | 2 |
| | | Variola | 1 |
| | Chronic | Purpura | 2 |
| | | Psoriasis | 1 |
| | | Urticaria | 3 |
| Various Parts,—Muscles, Tendons, Joints, &c. &c. | | Rheumatism, Acute | 5 |
| | | ——— Chronic | 32 |
| | | Gout | 1 |

* The arrangement adopted in this Table is similar to the one I made use of in the Report of the Infirmary for Children, and, I am aware, is liable to many objections. No apology, however, is necessary for discontinuing the uninteresting alphabetical catalogue of diseases usually adopted; and I can only regret that nothing better suggested itself to me.

***Diseases not easily referrible to particular Organs.***

| | | |
|---|---|---|
| Fevers | Continued | 13 |
| | Intermittent | 1 |
| Dropsies | Anasarca | 4 |
| | Ascites | 3 |
| | Hydrothorax | 1 |

Of the above cases, only five are known to have proved fatal:—Apoplexy, 1; Pericarditis, 1; Phthisis, 3.

*Apoplexy.*—I was requested, on the forenoon of the 7th ult. (August,) to visit a woman, aged forty-eight, residing in Great Wild-street. I found her in a state of stupor, from which she could not be roused; the pupil very much dilated, undulating slightly on the application of a lighted candle, but refusing to contract permanently; the breathing apparently natural, not at all stertorous; pulse frequent and small; skin warm and covered with clammy sweat. The account given of her was, that she had been a person of irregular habits, subject to occasional pain and giddiness in the head; that, finding herself more than usually indisposed on the preceding evening, she had taken some port wine, which potation had been repeated, the day I saw her, at six o'clock in the morning, when her husband went to work. On returning at nine, he found her unable to speak, although she seemed sensible to external objects, following the persons in the room with her eyes, and manifesting that she understood them. The stupor continued to increase gradually, and she died at five o'clock next morning; cupping, blisters, stimulating glysters, &c. having been employed in vain.

On examining the body the day after her death, in the presence of the pupils and apothecary to the Dispensary, particular attention was given to the state of the brain. The calvarum was unusually thick, and the furrows on its internal surface, from the impression of blood-vessels, were more numerous and deeper than common. The surface of the brain did not present any unnatural degree of turgescence; the upper part of the left hemisphere had some star-like points, from the florid injection of minute vessels; and, where the membrane passes from one convolution to another, there was slight watery effusion beneath it. The ventricles contained about three drachms of fluid on each side, and the plexus choroides were almost perfectly colourless, that on the left side having five or six little transparent bladders upon it, about the size of small peas. Careful examination led to the discovery of no other unnatural appearance. The contents of the thorax were sound. The liver had formed slight bands of adhesion with the diaphragm, and was rather softer than usual; the same was observed of the spleen. The bladder was very large, and contained about two pints of urine; (the attendants assured me she had made water regularly to the period of her death.) The uterus, unimpregnated, was about three-fourths of an inch thick, and felt as if cartilaginous.

The case of *Pericarditis* occurred in a girl of fourteen: she had laboured for six months under pain referred to the region of the heart, with extreme anxiety, for which bleeding, digitalis, and the usual routine practice had been employed. The pericardium was adherent throughout to the heart, from which it could only be separated by careful dissection. Round the roots of the great vessels was an effusion, three-quarters of an inch thick, transparent, of a pale straw colour, and having extremely minute blood-vessels ramifying through it in a very beautiful manner.

Of the cases of *Phthisis*, two were not examined; the other presented nothing remarkable.

### MEDICAL AND PHYSICAL INTELLIGENCE.

